# Depressive Symptoms, Self-Esteem and Perceived Parent–Child Relationship in Early Adolescence

**DOI:** 10.3389/fpsyg.2016.00982

**Published:** 2016-06-28

**Authors:** Alessandra Babore, Carmen Trumello, Carla Candelori, Marinella Paciello, Luca Cerniglia

**Affiliations:** ^1^Laboratory of Dynamic Psychology, Department of Psychological Sciences, Health and Territory, Università degli Studi “G. d’Annunzio” Chieti-PescaraChieti, Italy; ^2^International Telematic University UninettunoRome, Italy

**Keywords:** depression, self-esteem, early adolescence, mother–child relations, father–child relations

## Abstract

**Aims:** Early adolescence represents a critical developmental period both from a psychological and a psychopathological point of view. During this period, one of the most common disorders that frequently arise is represented by depression, that tends to become chronic and may produce many subsequent psychosocial impairments. The present study aimed to analyze characteristics of depressive symptoms in an Italian sample of early adolescents, and to explore their connections with self-esteem levels and perceived maternal and paternal emotional availability.

**Methods:** 594 adolescents (50% females) with a mean age of 12.11 years (*SD* = 0.98) were administered the Children’s Depression Inventory, the Rosenberg Self-Esteem Scale, and the maternal and the paternal forms of the Lum Emotional Availability of Parents.

**Results:** Findings highlighted a slightly higher, though not statistically significant, level of depressive symptoms in girls than in boys. Regression analysis showed that, as far as predictors of depression, self-esteem was the most relevant one, followed by maternal and paternal emotional availability.

**Conclusion:** Our results strongly suggested to plan intervention programs aimed at monitoring early adolescents’ self-esteem and supporting relationship with both parents, in order to prevent the emergence of depressive symptoms.

## Introduction

Early adolescence is considered a critical developmental period for exploring relations among parents, children, and the environment. The onset of adolescence, with the broad range of physical, emotional, and cognitive changes occurring, represents an important turning point in the individual’s development, not only from a psychological point of view, but also with regard to the emergence of psychopathology. One of the most common disorders that frequently arise in this period is represented by depression, that tends to become chronic and forecasts many subsequent psychosocial impairments (Yap et al., 2014), such as marked withdrawal and increased risk of suicide. Hence, the need to deepen its study, analyzing the different aspects that may serve as risk or protective factors.

Within this framework, several studies investigated the interaction between age and gender on depressive symptoms. Most research on this topic found no differences between males and females during childhood (e.g., Twenge and Nolen-Hoeksema, 2002; Babore et al., 2013); the effect of gender on depression seems to appear during adolescence, with a significantly higher level of depressive symptoms among girls than in male peers (Twenge and Nolen-Hoeksema, 2002; De Rubeis and Hollenstein, 2009; Masip et al., 2010; Piko and Balázs, 2012; Cerniglia et al., 2014). This pattern has been cross-culturally confirmed with a few exceptions: some Authors did not detect gender differences in adolescence (Helsel and Matson, 1984; Samm et al., 2008), while others found a higher depressive level in boys than in girls (Kovacs, 1992; Almqvist et al., 1999).

Referring to the Italian context, the few studies that analyzed gender differences in children’s and adolescents’ levels of depressive symptoms pointed out higher scores among girls when compared with boys: this result was confirmed by Poli et al. (2003) on a 8–17 year sample and by Pantusa et al. (2006) on 776 Italian students ranging between 11 and 18 years. A higher proportion of girls reporting depressive symptoms than boys was highlighted by Santinello and Vieno (2006) in a sample of 11-, 13-, and 15-year-old students.

Among risk and protective factors associated with depressive symptomatology during adolescence, self-esteem (e.g., Orth et al., 2014) and relationship with parents (e.g., Yap et al., 2014) play a relevant role.

As far as the relationship between self-esteem and depression, many studies have underlined significant connections between the two constructs in adolescence. In particular, adolescents’ self-esteem has been analyzed in relation to various fields such as parental and peer relationships, academic success, physical changes, and – consistently with our research topic – depressive experience. Low self-esteem may be connected to a higher likelihood of emerging problems such as depression, suicidal thoughts, adolescent pregnancy, eating disorders, and difficulty in social relationships (Emler, 2001; Yap et al., 2014). Among the psychological vulnerability factors, self-esteem plays an important role. According to several theories of depression, negative self-representations confer vulnerability to depression (Beck, 1967; Brown and Harris, 1978; Beck and Steer, 1987); in fact, low levels of self-esteem may exacerbate the effects of other vulnerability factors in the development of depression in children and adolescents (Abela, 2002; Southall and Roberts, 2002; Abela and Payne, 2003; Adams et al., 2007; Cimino et al., 2015). The relationship between self-esteem and depression has been analyzed in several longitudinal studies: all indicate that self-esteem may be considered as a predictor of depression, and not vice versa (Orth and Robins, 2013).

At the same time, examining the role of familial factors, previous studies have shown how adolescents’ functioning is deeply related to their perception of their parents’ behavior and to the quality of their interactions with them. Bosco et al. (2003) underlined some differences connected with gender: sons showed higher externalizing problems if they perceived lower levels of maternal acceptance, while daughters showed higher internalizing problems in relation to paternal depression and anxiety. Other researchers focused specifically on the presence of depressive symptoms in adolescence, exploring, at the same time, the characteristics of the relationships developed with parents. Branje et al. (2010) examined “longitudinal bidirectional effects” between adolescent’s relationship with parents and depression, underlying that older adolescents reported more depressive symptoms and lower quality of relationship with fathers and mothers. A more recent study (Piko and Balázs, 2012) explored the role of authoritative parenting style and other family variables in adolescents’ depressive symptomatology: these data support a negative association between authoritative parenting style and adolescent mood problems. In their research on a sample of late adolescents, McKinney et al. (2008) found that warm, authoritative parenting was associated with positive adjustment (valued in terms of depression, anxiety, and self-esteem): specifically, their emotional adjustment was related to perceived maternal parenting, but not to perceived paternal parenting. Authors strongly recommended to take into account the gender of both parents and to consider earlier developmental time periods in future studies.

As previous literature highlighted, early adolescence may be considered a critical phase, during which first psychopathological signs begin to appear; hence, our study specifically addressed this life stage, focusing on depressive symptomatology and its risk and protective factors, with the wider purpose to plan preventive interventions. Therefore, our general aim was to explore characteristics of depressive symptoms in early adolescence, according to gender and age, analyzing their connections with self-esteem and perceived parental (both maternal and paternal) emotional availability.

Focusing on gender differences, in accordance with findings of previous literature, we expected that in our sample of Italian adolescents, females should show higher levels of depressive symptoms than males. We also expected that depressive mood might increase with age.

As regards the relationship between depressive symptoms and self-esteem, we hypothesized that, independently from gender, self-esteem might play a decisive role in our sample, as indicated in former research from other countries.

Even if previous studies overall underlined the importance of the relationship with parents on the development of depressive symptoms, results of research that took into account parental gender differences were inconclusive. For this reason, in relation to our aim to analyze the impact of the perceived quality of relationship with parents on early adolescents’ depressive symptoms, we expected that parental emotional availability may affect children’s depression mood but – given the inconsistency of previous research – we did not formulate hypotheses on the differential impact of mothers and fathers.

## Materials and Methods

### Participants

The participants of the present study were 594 adolescents of several middle schools located in central Italy.

The sample was equally distributed by gender: 297 males (mean age = 12.12 years; *SD* = 1.07) and 297 females (mean age = 12.09 years; *SD* = 0.90), whose ages ranged from 10 to 13.9 years, with a total mean age of 12.11 years (*SD* = 0.98).

The mean age was 45.93 years (*SD* = 5.41; range 28–73) for fathers and 42.56 years (*SD* = 4.94; range 29–58) for mothers. Intact families made up 87.88% of the sample.

The families’ educational status placed at a middle level: a small percentage of subjects reported that parents have completed only the elementary school (0.97% of fathers and 0.19% of mothers); 25.24% of fathers and 19.78% of mothers completed middle school; 40% of fathers and 43.66% of mothers reached a high school diploma; 22.14% of fathers and 26.12% of mothers achieved a university degree. About one tenth of children was not able to answer the question about the level of education in relation to fathers (11.65%) and mothers (10.25%).

### Procedure

The present study has been realized according to the Ethics code of the Italian Association of Psychology (AIP). Before the research start, we asked permission to the Headmasters of the schools, and contacted all the parents of the students, so that they were well-informed about our project. In fact, written informed parental consent was obtained for all participants. Detailed information on how to complete the questionnaires were provided to participants. The administration took place in the classroom during the school hours and lasted about 25 min for each class; most students showed willingness to participate. All data have been anonymously collected and every student has been given a codename.

### Measures

#### Socio-Demographic Questionnaire

The first part of the questionnaires set comprised a socio-demographic sheet developed in order to collect data about participants’ age and gender, composition of their nuclear family, parents’ age and educational level.

#### Children’s Depression Inventory

The *CDI* (Kovacs, 1992) can be considered one of the most frequently used instruments for measuring the presence of depressive symptomatology through behavioral, emotional and cognitive aspects in children and adolescents (age range: 8–17 years). It is a 27-item self-report questionnaire, developed specifically for young people, modeled on the Beck Depression Inventory (Beck and Steer, 1987), the well-known depression questionnaire for adults.

Items are scored along a three-point scale from 0 to 2: 0 means that the symptom is not present, 1 that the symptom is present and mild, and 2 that the symptom is present and marked. The total score may range from 0 to 54; higher scores reflect a higher level of depressive symptoms. Following the suggestion by Authors of the Italian version, a cut-off of 19 can be considered a “pathological threshold” (Camuffo et al., 1988, p. 16); hence, we referred to this score in order to evaluate the incidence of depressive symptomatology in our sample. Many international studies have confirmed adequate internal consistency of the CDI and test–retest reliability, as well as convergent and discriminant validity (Kovacs, 1992). In the present research we have used the aforesaid Italian version, which showed in our sample an adequate internal consistency, with a Cronbach α’s coefficient of 0.84.

#### Rosenberg Self-Esteem Scale

The *RSES* (Rosenberg, 1965) is used for measuring self-esteem as an overall concept. It has been used with adults too, even if it was developed as an instrument for teenagers. Involved subjects are asked to evaluate their level of agreement or disagreement about 10 statements, using a four-point Likert scale (from 4 = “Strongly agree”, to 1 = “Strongly disagree”), with the total score ranging between 10 and 40. These items explore the individual’s satisfaction with himself/herself, and positive feelings. The higher the score, the better the self-image. We have used for this research the Italian validation by Prezza et al. (1993) of the RSES, that has shown good psychometric properties both in adolescence and adulthood (age range 15–60 years). In our study the Cronbach alpha’s coefficient was acceptable (α = 0.74).

#### Lum Emotional Availability of Parents

The *LEAP* (Lum and Phares, 2005) is a self-report questionnaire aimed at exploring adolescents’ perception of the emotional availability of parents (age range: 9–25 years). It consists of two forms (maternal and paternal), which permits to evaluate separately these two kinds of bonds. Each form consists of 15 items, rated according to a six-point Likert Scale (from 6 = “Always” to 1 = “Never”).

The total score, obtained by summing all the single items, ranges from a minimum of 15 to a maximum of 90 for every parent, with higher score indicating a higher level of emotional availability. The factor analysis of the Italian version of the LEAP seems to confirm its one-dimensional structure (Babore et al., 2012; Babore et al., 2014). In our study, the internal consistency of this instrument was found to be adequate with a Cronbach α’s coefficient of 0.90 for the maternal form and 0.94 for the paternal one.

### Data Analysis

The variables normality was preliminarily ascertained and correlation analyses were carried out on total sample. Thus, we began analyses by examining gender differences on study variables. *t*-Test was specifically used to test differences between males and females on depressive symptomatology, self-esteem, and parental emotional availability, also considering the Bonferroni correction, in order to control the multiple comparison issues that might lead to a type I error, i.e., to consider an effect as statistically significant when it is not (Cramer et al., 2016). A measure of the effect size was also determined through the Cohen’s *d*, with the following interpretation of results: *d* = 0.2 small, *d* = 0.5 medium, and *d* = 0.8 large effect size (Cohen, 1988). Finally, a multiple linear regression was conducted to test the explicative power of age, gender, self-esteem, and parents’ emotional availability on depressive symptomatology. Moreover, age × gender, gender × self-esteem, gender × parents’ emotional availability, age × self-esteem, and age × parents’ emotional availability interactions were examined in the regression model. Before performing regression analysis we have centered the age by subtracting mean-age from its every value. Finally Tolerance and the Variance Inflation Factor were checked to detect collinearity problems (Pedhazur, 1997). All statistical analyses were performed using SPSS v.15.0.

## Results

### Descriptive Statistics

As shown in **Table 1**, preliminary descriptive statistics were performed for all variables.

**Table 1:** Descriptive statistics.

	Mean	*SD*	Skewness	Kurtosis	1	2	3	4	5
**(1)** Age	12.11	0.98	0.024	-0.871		-0.040^b^	0.130^a^	-0.073^b^	-0.139^a^
**(2)** Depressive symptomatology	9.54	6.27	0.95	0.71	0.255		-0.58	-0.39	-0.22
**(3)** Self-esteem	28.85	4.51	-0.44	-0.18	-0.270	-0.65		0.38	0.15
**(4)** Maternal emotional availability	71.02	12.35	-0.85	0.64	-0.133^a^	-0.43	0.44		0.53
**(5)** Paternal emotional availability	65.95	16.23	-0.94	0.79	-0.154^a^	-0.36	0.31	0.52	


*Correlation coefficients are all significant *p* < 0.001, except ^a^*p < 0.05* and ^b^not significant. Correlation coefficients above the diagonal refer to the male sample, those that are below diagonal refer to the female sample.*

The CDI mean score was 9.54 (*SD* = 6.28; range 0–34). In our sample, 9.26% (*N* = 55) of participants exceeded the cut-off of 19 that can be considered a “pathological threshold” according to the Italian manual (Camuffo et al., 1988) of the CDI.

Moreover, correlational analyses showed that depressive symptomatology was significantly and negatively related to self-esteem and parental emotional availability; it was also significantly associated with age but only among females; maternal emotional availability, paternal emotional availability and self-esteem were positively associated among them.

### Gender Differences

*T*-test showed that males and females significantly differed on self-esteem and maternal emotional availability, but not on paternal emotional availability (**Table 2**). Instead, the gender difference we found in depression might be a type I error because of the multiple comparison issues; in fact, with the Bonferroni adjustment (*α* = 0.0125), this difference was no longer significant with regard to depression. In addition the effect size relieved for depressive symptomatology was small.

**Table 2:** *T*-test: gender differences.

	Females		Males		Student’s *t*	*Sig.*	Cohen’s *d*
					
	Mean	*SD*	Mean	*SD*	*t*_(df)_		
Depressive symptomatology	10.06	6.67	9.02	5.82	2.012_(592)_	*0.045*	0.17
Self-esteem	28.23	4.68	29.46	4.26	-3.357_(592)_	*0.001*	0.27
Maternal emotional availability	72.75	11.72	69.30	12.75	3.438_(592)_	*0.001*	0.28
Paternal emotional availability	65.49	17.05	66.41	15.39	-0.691_(586)_	*0.490*	0.06


### Regression Models

The regression analysis showed that all interactions (age × gender, gender × self-esteem, gender × parents’ emotional availability, age × self-esteem, and age × parents’ emotional availability) were not significant. Thus, the interactions were not considered in the final regression model. The final analysis attested that both self-esteem and parental emotional availability significantly predicted depression (**Table 3**). Moreover, Tolerance and Variance Inflation Factors did not show collinearity problems. The standardized beta coefficients highlighted that high levels of self-esteem and parental emotional availability predicted low levels of depressive symptomatology, while gender and age were not significant predictors. However, if only age and gender were considered in the regression model, results indicated that they significantly affect depression (*R*^2^ = 0.02, *p* = 0.005; gender standardized β = -0.09, *p* = 0.03; age standardized β = 0.10, *p* = 0.01). Thus it is possible that the influence of gender and age on depression is mediated by self-esteem and parental emotional availability.

**Table 3:** Regression analysis for depressive symptomatology.

	Depression	Collinearity Indices	
		
	β	Tolerance	VIF
Gender	-0.03	0.93	1.07
Age	0.05	0.98	1.02
Self-esteem	-0.55^∗∗∗^	0.82	1.22
Maternal emotional availability	-0.14^∗∗∗^	0.63	1.58
Paternal emotional availability	-0.09^∗∗^	0.72	1.38
*R*^2^	0.43^∗∗∗^	
AR	0.43^∗∗∗^	


*β = beta coefficients; *R*^2^ = Total *R* Square; AR = Adjusted *R*^2^; ^∗∗∗^*p* < 0.001; ^∗∗^*p* < 0.01.*

## Discussion

The present study aimed to explore characteristics of depressive symptoms in early adolescence, according to age and gender, analyzing their connections with self-esteem and perceived parental emotional availability.

Studies on the onset of depressive symptoms highlighted that the first years of adolescence may be considered a critical phase. In light of this finding, we focused on this period in order to detect features of depression in a sample of Italian adolescents. The CDI mean score found in our sample was comparable to the one observed in other studies using the same tool, carried out on Italian (Poli et al., 2003; Pantusa et al., 2006), Estonian (Samm et al., 2008), Hungarian (Piko and Balázs, 2012) and extra-European (Twenge and Nolen-Hoeksema, 2002) samples. Also the percentage of adolescents reporting a depressive level above the risk threshold was consistent with results of other studies using this same self-report questionnaire in Italy (Frigerio et al., 2001; Poli et al., 2003) and in other countries (Masip et al., 2010). These results constitute a cause for concern since they indicate that depressive symptomatology represents a fairly common phenomenon among adolescents.

Results of previous research focusing on gender differences in depressive symptomatology were quite inconsistent: although most studies observed a higher depressive level in females than in males (Poli et al., 2003; Piko and Balázs, 2012), other studies did not detect gender differences (Samm et al., 2008) or found males scoring higher than females (Kovacs, 1992). Findings of our research revealed only a slightly higher level of depressive symptoms in girls, as compared with boys. Some theorists argued that the physiological changes caused by puberty may increase vulnerability to depression (e.g., Angold et al., 1998), primarily among girls. Within this topic, Pantusa et al. (2006) highlighted the differential impact that pubertal changes exert on the two genders: while males generally express more satisfaction with somatic changes associated with puberty, females tend to feel increased discomfort. This hypothesis seems to be confirmed also by the decreased levels of self-esteem in girls starting from early adolescence (Rawana and Morgan, 2013), a result found also in our sample. However, contrary to our expectations, our data did not reveal a significant difference on depression between males and females. A possible reason to explain this finding might be sought in our sample age: in fact, we considered only the earliest years of adolescence, when gender differences on depressive mood have not yet fully arisen. A support to this hypothesis seems to come from the study of Samm et al. (2008) who, considering a sample of late childhood and early adolescence children, did not observe differences between males and females.

With regard to age, through a correlational analysis we found that depressive mood increased with age but only among girls. However, in the regression analysis both age and gender became not significant when other predictors were introduced in the model. This finding let us to hypothesize that the influence of gender and age on depression is mediated by self-esteem and parental emotional availability. Anyway, these results need to be confirmed by further studies, with a longitudinal design, that more appropriately allow the use of a mediation model (Maxwell and Cole, 2007).

As regards the second aim of our study, we analyzed the link between depressive symptoms and self-esteem. Our results support the hypothesis that self-esteem and depression are strongly correlated. In our research, in fact, self-esteem was the most significant predictor for depressive symptomatology during early adolescence. This relation between low self-esteem and depression is consistent with the literature on clinical and non-clinical populations (Joiner et al., 1999; Emler, 2001; Klein et al., 2011; Orth and Robins, 2013). The study of MacPhee and Andrews (2006) about several risk factors for depression in early adolescence, showed that self-esteem accounted for the majority of explained variance in depressive scores. According to the vulnerability model (Beck, 1967; Metalsky et al., 1993), low self-esteem influences the onset of depression: individuals with negative beliefs about themselves are at risk for developing depressive symptoms.

Self-esteem is relevant for several personal and social life outcomes and, as mentioned above, a low level of self-esteem may serve as a risk factor for depression in adolescence and young adulthood (Orth et al., 2008). In a recent research, Steiger et al. (2014), studying the long-term effects of self-esteem development, found that individuals who had a low self-esteem during adolescence exhibited more depressive symptoms two decades later, in adulthood. In addition, another line of research demonstrated that for early adolescents, improving their self-esteem is important in avoiding depression (Jun et al., 2013).

Finally, we have analyzed connections between depression and emotional quality of relationship with parents. As well known, a positive quality of parent–adolescent relationship, security of attachment and parental availability may serve as protection (Cimino et al., 2013). A first result was the importance of a positive relationship with both parental figures, with a slightly greater importance of the maternal emotional availability over the paternal one. Existing literature mainly highlighted the importance of the mother: Besser and Blatt (2007) underlined that she tends to be the preferred attachment figure in western cultures. McKinney et al. (2008), presenting the outcomes of their research, observed that perceived paternal parenting does not have a direct relationship to late adolescents’ emotional adjustment. They hypothesized that, even if maternal and paternal roles have changed over time, maternal parenting continues to play a more relevant role than paternal parenting in late adolescent adjustment. In our data about early adolescence, although the mother represented the most significant figure for girls and boys, the father also seemed to be very important for both daughters and sons. Consistent with our result about the absence of a very significant difference on depression between males and females, we also found that regardless of gender, the relationship with both parents may play a relevant role in emotional adjustment during early adolescence. Moreover, since no interactions between gender and study variables were observed, our data suggested that the impact of self-esteem and parental emotional availability had similar weight for both female and male subjects.

Results of the current study must be considered in the light of some methodological limitations. First, the use of a self-report instrument for depression, without a clinical assessment, did not enable us to formulate a specific diagnosis. Second, the used tool, though one of the most common worldwide, was not able to detect depressive equivalents, namely behaviors masking a depressive suffering (as, for example, antisocial behaviors, alcoholism, and drug abuse) that have been more frequently found among depressed males than depressed females (Cochran and Rabinowitz, 2000). Third, the cross-sectional nature of the current research limited the possibility to draw meaningful conclusions about the cause-and-effect relationship between depressive symptoms and their correlates. Further studies could try to address these limitations, also widening the age range of the sample in order to include middle and late adolescence; in addition, for future research it would be interesting to explore the dyadic interconnections between self-esteem and parental emotional availability in predicting depression.

Notwithstanding these limitations, some strengths can be highlighted: first, the exploration of adolescents’ perceptions of both maternal and paternal emotional availability; in fact, previous research investigating associations between parental availability and emotional adjustment in adolescence mainly focused on one parental figure (mother or father) or both, but involving only female adolescents (Steinberg and Davila, 2008; Cordero and Israel, 2009; Demidenko et al., 2015). Another strength of the current study was represented by the collection of data on Italian adolescents, considering that most of the existing literature on this topic comes from either North American or North European samples: in this perspective, our study gives a contribution to deepen the transcultural knowledge about risk and protective factors of adolescents’ depression.

## Conclusion

In our research some consistent findings emerged on the importance of early detection of risk conditions for the onset of depressive symptomatology. In fact, given the reorganization that takes place during adolescence, it is in this period that individuals typically start to build up their identity and extra-familial relationships: self-esteem may play a crucial role in the development of these internal processes. Adolescents are prompted to increase introspection in order to find out who they really are and how they are perceived by their environment (Steinberg, 2005). In this phase, there are new academic and social demands, transformations in self-perceptions and reorganizations in parent–child relationships (Steinberg, 1987; Savin-Williams, 1990).

Following these considerations and the findings of our study, it is strongly suggested to plan early intervention programs – capable to involve both males and females – aimed at monitoring early adolescents’ development, consolidating their self-esteem and supporting relationship with parents, in order to prevent the emergence of depressive symptoms.

## Author Contributions

All authors listed, have made substantial, direct and intellectual contribution to the work, and approved it for publication.

## Conflict of Interest Statement

The authors declare that the research was conducted in the absence of any commercial or financial relationships that could be construed as a potential conflict of interest.

## Abbreviations

CDI: Children’s Depression Inventory
LEAP: Lum Emotional Availability of Parents
RSES: Rosenberg Self-Esteem Scale
